# Long-term efficacy of endovascular vs open surgical repair for complicated type-B aortic dissection: a single-center retrospective study and meta-analysis

**DOI:** 10.1590/1414-431X20165194

**Published:** 2016-05-31

**Authors:** Y. Zhu, B. Wang, Q. Meng, J. Liu, S. Zhai, J. He

**Affiliations:** 1 Department of Cardiothoracic Surgery, Kaifeng Central Hospital, Kaifeng, China; 2 Department of Cardiothoracic Surgery, Henan Provincial People's Hospital, Zhengzhou, China

**Keywords:** Aortic dissection, Open surgical repair, Type-B aortic dissection, Endovascular aortic repair

## Abstract

This study aimed to evaluate the long-term survival and risk factors of traditional open surgical repair (OSR) *vs* thoracic endovascular aneurysm repair (TEVAR) for complicated type-B aortic dissection (TBAD). A total of 118 inpatients (45 OSR *vs* 73 TEVAR) with TBAD were enrolled from January 2004 to January 2015. Kaplan-Meier curves and Cox proportional hazards analysis were performed to identify the long-term survival rate and independent predictors of survival, respectively. Meta-analysis was used to further explore the long-term efficacy of OSR and TEVAR in the eight included studies using Review Manager 5.2 software. An overall 10-year survival rate of 41.9% was found, and it was similar in the two groups (56.7% OSR *vs* 26.1% TEVAR; log-rank P=0.953). The risk factors of long-term survival were refractory hypertension (OR=11.1; 95%CI=1.428-86.372; P*=*0.021] and preoperative aortic diameter >55 mm (OR=4.5; 95%CI=1.842-11.346; P*=*0.001). Long-term survival rate did not differ significantly between OSR and TEVAR (hazard ratio=0.87; 95%CI=0.52-1.47; P*=*0.61). Compared with OSR, TEVAR did not show long-term advantages for patients with TBAD. Refractory hypertension and total aortic diameter >55 mm can be used to predict the long-term survival of TBAD in the Chinese Han population.

## Introduction

People with type-B aortic dissection (TBAD) with complications such as rupture or organ ischemia often require emergency surgery (1). Previous studies have shown that increased complexity due to organ perfusion in aortic dissection, aortic rupture, refractory hypertension, persistent pain, and other factors may increase TBAD mortality (2). Therefore, appropriate treatment for patients with acute aortic dissection is critical for therapy efficiency and survival.

Artificial blood vessel transplantation has been successfully used to repair thoracic aneurysms and descending aortic dissection since first published (3), although traditional open surgery repair (OSR) has been considered the preferred treatment for TBAD (4). Surgical resection of the thoracic aorta is still associated with high mortality and morbidity from complications, such as neurological ones, despite remarkably improved operative techniques (5). However, given that endovascular stent implantation was successfully used to treat abdominal aortic aneurysm in 1991 (6) and TBAD in 1999 (7), thoracic endovascular aortic repair (TEVAR) has been widely used and has attained global expansions.

Various studies have indicated that TEVAR, which shows some advantages such as being less traumatic, having a low fatality rate, having a reduced surgical time, and less blood transfusion requirement (8), was beneficial in increasing the 30-day survival rate in patients with aneurysm (9). Therefore, this technique provides an attractive and less invasive alternative therapy for TBAD. However, complications with TEVAR, such as endoleaks and graft transplantations, may occur more easily than with OSR (10), and the uncertain long-term results for TEVAR have remained a special concern. In addition, studies on long-term survival rate of TEVAR and OSR are inconsistent (11,12). Hence, in the present study, we aimed to evaluate the long-term efficacy and risk factors of traditional OSR *vs* TEVAR for complicated TBAD.

## Material and Methods

### Subjects

A total of 118 TBAD inpatients (45 and 73 cases treated with OSR and TEVAR, respectively) who received professional treatment in Kaifeng Central Hospital during January 2004 to January 2015 were included in the retrospective study. TBAD was diagnosed through magnetic resonance angiography (MRA) or CT angiography (CTA) by at least two experienced clinicians. Patients who presented with dissection because of iatrogenic or traumatic reasons were excluded, as well as those who had Marfan syndrome or dissection combined with severe chronic diseases, such as cancer. The median follow-up of the study was 56 months. Finally, 22 patients were either lost or refused to be followed-up. The study was conducted in accordance with the Helsinki Declaration and all participants signed informed consents. The study was approved by the ethics committee of the Kaifeng Central Hospital.

### Therapeutic approach

Of the 45 patients in OSR group, 8 underwent partial sternotomy and lateral thoracotomy, 15 had full sternotomy, and 22 had lateral thoracotomy. Among these cases, 18 received descending aorta replacement, 14 received both aortic arch and descending aorta replacement, 9 received both partial aortic arch and descending aorta replacement, and 4 received both thoracic and abdominal aorta replacement. In the TEVAR group (73 cases), the reasons for intervention included aneurysms, anatomical defects, penetrating ulcers, or sham aneurysms. Eighty-three percent were considered high risk for open surgery because of old age or poor performance status. The treatment area included ascending aorta (n=2), distal arch (n=20), and proximal (n=40) or distal descending aorta (n=45). Devices were delivered by femoral (79.9%), retroperitoneal-iliac (16.1%), or carotid exposure (4%). Devices used included Gore TAG (WL Gore & Associates, Inc., USA, n=30), Medtronic Talent or Valiant (Medtronic, Inc., USA, n=35), Zenith (Cook, USA, n=3), Cook TX2 (Cook, n=15), and custom-fabricated (n=3). The decision for the appropriate therapeutic approach was based on patient desire, as well as the preoperative evaluation of CTA/MRA results (13,14). Interventions were successfully conducted under guidance of digital subtraction angi-ography surveillance. All patients were transferred to the ICU for further care and treatment after OSR or TEVAR.

### Meta analysis - Literature search strategy

The terms "open", "repair/surgery", "endovascular", "dissection", and "type B" were used for a comprehensive search in Medline, EMBASE, Web of Science, EBSCO, and Springer for studies until May 2015. Bibliographies of the retrieved articles were hand-searched for additional potentially relevant studies.

### Selection criteria and data extraction

According to the Preferred Reporting Items for Systematic Reviews and Meta-analyses (PRISMA) guidelines, the following criteria were applied to determine whether articles would qualify for the analysis: 1) studies comparing TBAD outcomes treated with TEVAR *vs* OSR and 2) the reported long-term results must contain survival rates or survival curves. Furthermore, the excluded articles had to satisfy the following criteria: 1) the comparison was not conducted as TEVAR *vs* OSR; 2) presence of repeated samples or duplicate publications, and 3) the follow-up time was less than 5 years. A standardized form was used to extract data from the articles, including characteristics of study design, study population, demographics, and long-term survival rate. Term search was performed by authors Y. Zhu and S. Zhai, whereas data extraction was performed by Y. Zhu and J. Liu. Disagreements were handled with discussions and final consensus. All data were checked for internal consistency.

### Data analysis

For the retrospective study, data were statistically analyzed using SPSS 19.0 software (IBM, USA). The results are reported as means±SD. Student's *t*-test was used to compare continuous variables and Fisher or chi-square test were used to compare categorical variables. The Kaplan-Meier curve method was used to examine the long-term survival in OSR and TEVAR, and Cox proportional hazards analysis was performed to identify the independent predictors of survival. P<0.05 was considered to be statistically significant.

For the meta-analysis, patient characteristics and outcomes were entered into the database. If data for hazard ratio (HR), 95%confidence intervals (CI), and long-rank P value could not be directly acquired from articles or corresponding authors, the data were extracted from the figures of Kaplan-Meier survival curve and then transferred to the Engauge Digitizer 4.1 software (http://markummitchell.github.io/engauge-digitizer/) and to Excel (Microsoft, USA) (15) under the guidance of Guyot (16). Meta-analysis was then conducted using Review Manager 5.2 software (http://community.cochrane.org/). HR and 95%CI were calculated to evaluate survival rates. Heterogeneity of results across trials was calculated and assessed with *I* ^2^. When *I* ^2^>50%, heterogeneity was considered significant, and sensitivity and subgroup analyses were further performed. Publication bias was assessed through funnel plots. P≤0.05 was set as the threshold for statistical significance.

## Results

### Retrospective study

**Figure 1:**
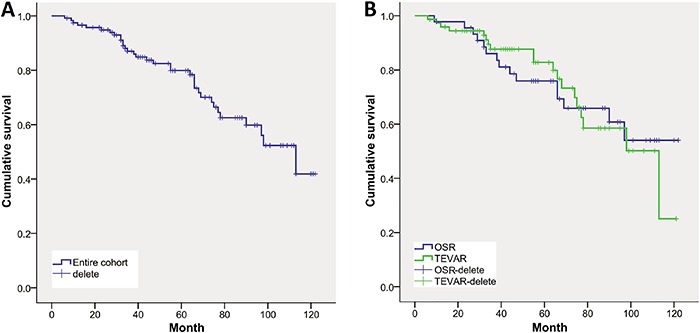
Kaplan-Meier analysis. *A*, The 10-year overall survival rate was 41.9% and the follow-up rate was 81.4%. *B*, No significant difference was found in the 10-year overall survival rate between open surgical repair (OSR) (56.7%) and thoracic endovascular aneurysm repair (TEVAR) (26.1%) in patients with type-B aortic dissection (log-rank, P*=*0.953).

The general clinical characteristics and preoperative management of the patients with TBAD are shown in Table 1. Patients in the OSR group were significantly younger than those in the TEVAR group (P*=*0.006). The TEVAR group had shorter surgical time (P*=*0.001), and the proportion of female patients in the TEVAR group was significantly higher than that of the OSR group (P*=*0.033). No significant difference was found in the preoperative aortic diameter between the two groups. There were no differences between the groups in number of malperfusion, shock and cardiopulmonary resuscitation. Crude mortality of the entire cohort was 31.36% (n=37), three of which were not caused by aorta-related events. Furthermore, Kaplan-Meier analysis showed that the estimated 10-year survival was 41.9% (Figure 1A). When stratified by treatment approach, patients in the OSR group had slightly higher 10-year overall survival rate than TEVAR (OSR 56.7% *vs* TEVAR 26.1%, log-rank *χ* ^2^=0.004, P=0.953, Figure 1B). Cox regression analysis revealed that refractory hypertension (P*=*0.021, OR=11.104, 95%CI=1.428–86.372) and preoperative aorta diameter of >55mm (P=0.001, OR=4.509, 95%CI=1.842–11.036) were the risk factors of long-term survival in the entire cohort (Table 2). The incidence of long-term aorta-related events, including endoleaks, aortic rupture, and acute type A dissection, did not significantly differ between TEVAR (10.9%) and OSR (11.1%) groups (*χ* ^2^=0.001, P=0.980). Meanwhile, the occurrence time to long-term aorta-related events was 39.63±37.51 months after TEVAR, and slightly earlier (28.00±20.03 months) for OSR (*t*=0.726, P=0.483). Table 3 illustrates the late treatment failures of these 13 patients along with re-intervention and outcomes.

**Table:**
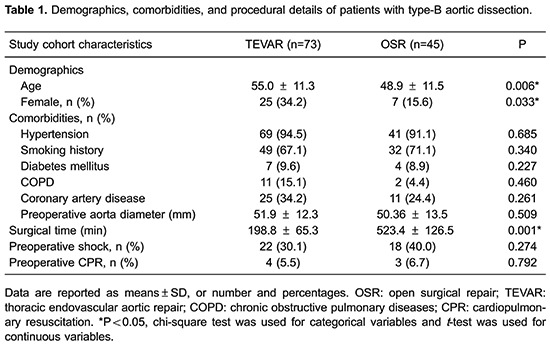

**Table:**
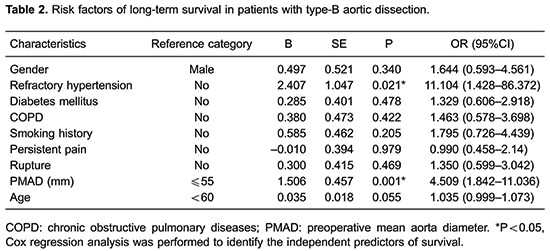

**Table:**
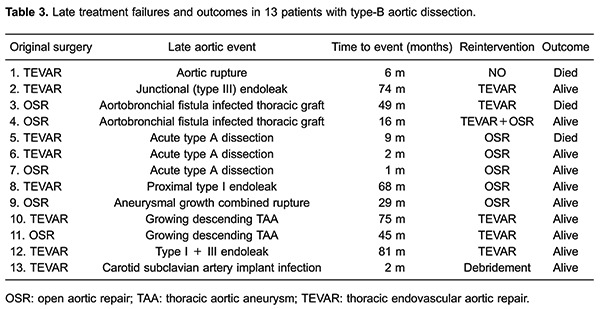

### Meta-analysis study

**Figure 2:**
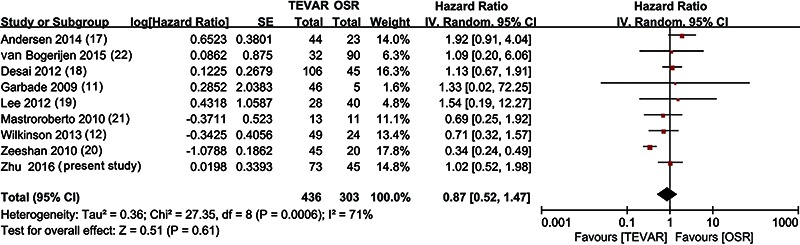
Meta-analysis of long-term overall survival rate between open surgical repair (OSR) and thoracic endovascular aneurysm repair (TEVAR) in patients with type-B aortic dissection.

A total of 2,695 abstracts were identified for screening, but only eight studies (11,12, 17-22) met the inclusion criteria. Finally, these eight studies and the present study involving a total of 739 patients (436 for TEVAR and 303 for OSR) were included in the meta-analysis. No significant difference was observed in the long-term overall survival rate between TEVAR and OSR (P=0.60, HR=0.87, 95%CI=0.52–1.98, Figure 2), although the former had a slight advantage. Sensitivity analysis revealed that these findings were preserved throughout the studies, except for the heterogeneous studies (11,19). Subgroup analyses stratified by nation or years of follow-up also showed that the long-term overall survival rate was similar in these two groups (P>0.05). In addition, funnel plot analysis identified that some biases existed in the meta-analysis.

## Discussion

Endovascular repair, especially TEVAR, is a less invasive method for TBAD treatment and has been considered an important progress in surgery. In this study, we evaluated the advantages of TEVAR and OSR by comparing these two techniques in TBAD treatment. Although the age and gender of patients were different, TEVAR had obvious advantages, such as shorter operating time and being less traumatic, which can be more suitable for elderly and female patients with difficulty in tolerating serious trauma from the surgery.

Hypertension may be the most common risk factorin patients with TBAD (23), and approximately 93.2% of the patients in this study were diagnosed with that disorder, implying that systemic vascular diseases may be associated with the pathogenesis of dissections. Hypertension, especially refractory hypertension, was found to be an independent predictor of late poor prognosis. Previous studies demonstrated that the rupture of aortic dissection is be the most frequent cause of death. At the same time, the maximized diameter of the dissecting aneurysm was associated with the increased risk of the rupture of aortic dissection (24), thereby making it an indirect cause of death. However, the present study revealed that preoperative aortic diameter of >55 mm was also a risk factor for long-term survival of TBAD and it has been previously identified as an unsuitable predictor for type A aortic dissection (25). These results suggest that the existence of refractory hypertension and aortic diameter >55 mm before surgery can be considered as risk predictors for long-term survival of patients with TBAD.

Abundant literature is available with regard to the adverse events after aortic stent graft is inserted into the chest (10,12), with the most common complication being endoleak, which is a serious sequela that may lead to secondary rupture of the aneurysm. The most important risk factor for endoleaks after TEVAR treatment of type B dissection was the existence of a short infrarenal neck. To maximize the neck length (anatomical requirements included ≥ 20 mm suitable proximal and distal neck length, and proximal and distal neck diameters of 20 to 42 mm), we used the distance between the origins of the left common carotid artery and the left subclavian artery (LSA), occluding the LSA when necessary. Endoleaks were observed in three patients after endovascular repair, which was forced after sealing the endoleaks with additional stent grafts. No clinical signs of malperfusion of the left arm or reperfusion of the false lumen (end leak) of the LSA were observed in these patients. In addition, the most alarming complication in this series was the retrograde type A dissection after implanting a stent graft that occurred in three patients who experienced refractory hypertension before surgery (2 in TEVAR *vs* 1 in OSR). This result suggests a pathophysiological mechanism between the two procedures. However, the time of the occurrence was not consistent, thereby suggesting a different cause, which also needs further study.

Malperfusion syndrome is reported in approximately 10% of patients with acute TBAD and typically leads to paraparesis or paraplegia, lower limb ischemia, abdominal pain, nausea, and diarrhea (5). Paraplegia after endovascular repair did not occur in the present study. The reason may be associated with the short grafts that were used in some cases to avoid covering the inter-costal artery, from which the Adamkiewicz artery originated, in the stent implantation. Therefore, it is possible to reduce the occurrence of adverse phenomena, such as lower limb ischemia or other malperfusion syndrome in the subsequent follow-up.

Several studies reported that patients treated with TEVAR had significantly higher short-term survival rate than those treated with OSR (26–28), whereas the long-term survival rates of TEVAR may not be higher than that of OSR. The 10-year overall survival rate for the entire cohort was 41.9%, which is similar to the result of an epidemiological study (29). However, the present study revealed that the 10-year overall survival rate in TEVAR was similar to that in OSR, although the latter had a slight advantage. This is consistent with a few previous studies (17,18,22), which demonstrated no long-term advantage in the treatment by TEVAR. Furthermore, the incidence and occurrence time of long-term aorta-related events, including endoleaks, aortic rupture, and acute type A dissection, were also similar between the two groups; approximately 89% were free from re-intervention. This treatment efficacy was also consistent with that of 5- and 20-year follow-up studies in American samples (12,22). However, the studies of Desai (18) and Di Luozzo (30) reported that the risk of re-intervention was significantly lower in OSR than in TEVAR. This inconsistency may be attributed to the characteristic of the samples, material, procedure, selection of grafts or the access site, as well as the preoperative medical condition of the patients.

Several unavoidable limitations existed in the present study. First, the restricted hospital capacity may impose limits to sample size, and some patients were lost during follow-up. Second, the quality of the studies included in the meta-analysis was limited, which may be caused by the retrospective experimental design. Third, the years of follow-up and nationalities of samples were inconsistent in the included studies, which may have been the source of heterogeneity. Fourth, the data of survival extracted from Kaplan-Meier curves and some unpublished negative results may have included biases, which were obviously present in the meta-analysis.

## Conclusion

Based on our results, the TEVAR does not have long-term advantages, such as survival rates, for patients treated with TBAD when compared with OSR. Both surgical techniques could be considered as suitable choices for the treatment of TBAD due to the similar long-term efficacy. Furthermore, refractory hypertension and total aortic diameter >55 mm can be used to predict the long-term survival of patients with the disorder in the Chinese Han population.
